# Design, Ground Testing and On-Orbit Performance of a Sun Sensor Based on COTS Photodiodes for the UPMSat-2 Satellite

**DOI:** 10.3390/s21144905

**Published:** 2021-07-19

**Authors:** Angel Porras-Hermoso, Daniel Alfonso-Corcuera, Javier Piqueras, Elena Roibás-Millán, Javier Cubas, Javier Pérez-Álvarez, Santiago Pindado

**Affiliations:** Instituto Universitario de Microgravedad “Ignacio Da Riva” (IDR/UPM), ETSI Aeronáutica y del Espacio, Universidad Politécnica de Madrid, Plaza del Cardenal Cisneros 3, 28040 Madrid, Spain; daniel.alfonso.corcuera@upm.es (D.A.-C.); javier.piqueras@upm.es (J.P.); elena.roibas@upm.es (E.R.-M.); j.cubas@upm.es (J.C.); javier.perez@upm.es (J.P.-Á.); santiago.pindado@upm.es (S.P.)

**Keywords:** UPMSat-2, sun sensor, space systems, attitude control, ADCS, on-orbit performance

## Abstract

This paper presents the development of the UPMSat-2 sun sensor, from the design to on-orbit operation. It also includes the testing of the instrument, one of the most important tasks that needs to be performed to operate a sensor with precision. The UPMSat-2 solar sensor has been designed, tested, and manufactured at the *Universidad Politécnica de Madrid* (UPM) using 3D printing and COTS (photodiodes). The work described in this paper was carried out by students and teachers of the Master in Space Systems (*Máster Universitario en Sistemas Espaciales*—MUSE). The solar sensor is composed of six photodiodes that are divided into two sets; each set is held and oriented on the satellite by its corresponding support printed in Delrin. The paper describes the choice of components, the electrical diagram, and the manufacture of the supports. The methodology followed to obtain the response curve of each photodiode is simple and inexpensive, as it requires a limited number of instruments and tools. The selected irradiance source was a set of red LEDs and halogen instead of an AM0 spectrum irradiance simulator. Some early results from the UPMSat-2 mission have been analyzed in the present paper. Data from magnetometers and the attitude control system have been used to validate the data obtained from the sun sensor. The results indicate a good performance of the sensors during flight, in accordance with the data from the ground tests.

## 1. Introduction

On 2 September 2020, the UPMSat-2 was launched as part of the Small Spacecraft Mission Service (SSMS) Proof of Concept (PoC) VEGA flight. This was the VV16 VEGA flight and represented a great challenge, as 53 satellites from 21 different customers were launched [1]. The UPMSat-2 is a 50 kg satellite designed, developed, and tested at the *Instituto Universitario de Microgravedad* “*Ignacio Da Riva*” (IDR/UPM) from *Universidad Politécnica de Madrid* (UPM). This satellite represents a successful project of space systems engineering that gathered university research and payloads from different companies (Bartington, Tecnobit, SSBV, Iberespacio, etc.). In addition, several academic projects were carried out within this project, which also transmitted a big impetus to the Master in Space Systems (*Máster Universitario en Sistemas Espaciales*—MUSE) at the *Universidad Politécnica de Madrid* (UPM) [2,3,4].

The UPMSat-2 project has promoted different research lines related to space systems engineering. Among them, it is possible to mention the work carried out by IDR/UPM Institute researchers related to: Attitude Determination and Control Subsystems (ADCSs) based on interaction with the Earth’s magnetic field; thermal control subsystems; structural analysis of spacecraft and space instruments/systems; software system for flight and ground segments; and spacecraft power subsystems (devoted to photovoltaic systems-solar cells/panels-performance, harness design, and Li-ion batteries performance).

Focusing on the UPMSat-2 ADCS, the satellite is magnetically controlled and spin stabilized. It is composed of [5,6]:

- SSBV magnetometers, to measure the orientation of the satellite in relation to the Earth’s magnetic field;
- ZARM Technik AG magnetorquers, that produce the torques to change the satellite’s attitude;
- A control law developed at IDR/UPM Institute that use the information from the magnetometers to order the magnetorquers action.

Two important research projects related to ADCS are planned to be carried out within the UPMSat-2 life program:

- The recalibration of the magnetometers based on the measurements of the Earth’s magnetic field carried out by the satellite;
- The validation of the COTS (Commercial Off-The-Shelf) photodiodes-based sun sensor designed, built, and tested for this mission at the IDR/UPM Institute.

The use of sun sensors as part of spacecraft ADCSs is not rare and, furthermore, it can be said that they are one of the most common sensors used on small satellites. They determine the satellite orientation in relation to the Sun. This information is transmitted to the On-Board Computer (OBC) that filters it, and then combines it with other data from different sensors (magnetic, Earth’s horizon, etc.) to determine the attitude of the satellite and improve the subsystem’s response [7,8,9,10,11].

In the present paper, a simple way to design, test, and operate a solar sensor based on COTS photodiodes is described. Of course, it is necessary to select the photodiodes and position them correctly, but that is only the first step. To have an accurate instrument, a proper response curve for each photodiode of the solar sensors is required. This is carried out by fitting the curve that relates the angle of incidence of the Sun to the response of each photodiode. Then, the information of all sensors together can be processed to obtain the direction of the sun. 

One of the main concerns when obtaining the response curve of the photodiodes is that these sensors perform differently depending on the light’s wavelength distribution (or spectrum). In the case of COTS photodiodes, it is not common for calibration information to refer to the solar spectrum in space. Instead, the calibration normally refers to the Sun irradiance on Earth. These results are not adequate, as due to the interaction with the atmosphere, the Sun irradiance received at Earth’s surface is not characterized by the same spectrum (AM1,5) as the satellite would receive in space (AM0) [12]. Most of the photodiode calibration techniques found in the available literature are based on the simulation of the AM0 spectrum [7,13,14], but this normally requires specific instrumentation and can increase the development cost of the solar sensor. As the objective of this work is to keep things simple, a great deal of effort has been put into simplifying a fitting process so as to not to depend on this specialized instrumentation.

The paper is organized as follows. In Section 2, the elements of the solar sensor are described. In Section 3, the testing set-up and the methodology are described. Results and validation are included in Section 4, whereas conclusions are summarized in Section 5.

## 2. Sun Sensor Design and Fabrication

**Figure 1:**
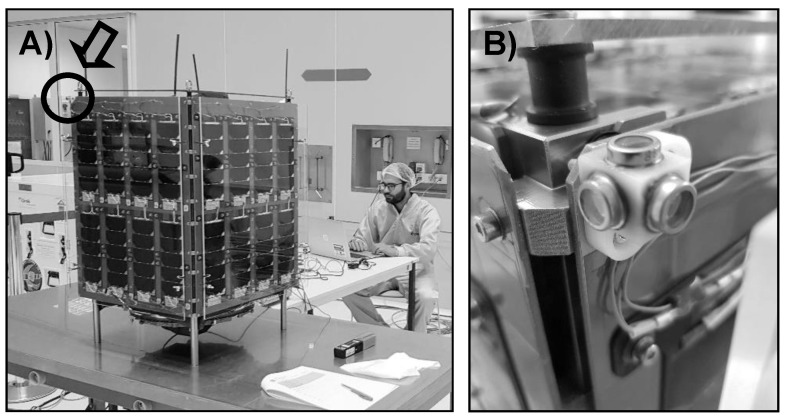
(**A**) UPMSat-2 at satellite at the Centre Spatial Guyanais of CNES (Kourou, French Guiana). February 2020. (**B**) SS1 sun sensor set, composed of three photodiodes. Pictures courtesy of Instituto Universitario de Microgravedad “Ignacio Da Riva”, Universidad Politécnica de Madrid.

In Figure 1, the sun sensor designed and fabricated for the UPMSat-2 in the IDR/UPM Institute is shown. The design methodology and the considerations taken into account during the manufacturing process of the sun sensor are described in this section.

### 2.1. Sun Sensor Physical Design

**Figure 2:**
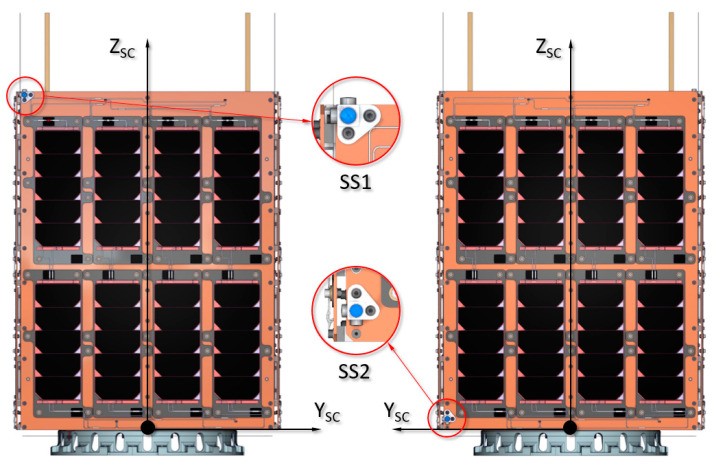
Front and rear view of the UPMSat-2 satellite showing the reference system and the position of the SS1 and SS2 sun sensor sets.

The UPMSat-2 satellite, whose geometric shape is an orthohedron with dimensions of 450 mm × 450 mm × 600 mm, is equipped with a sun sensor formed by two sets of photodiodes (SS1 and SS2) located at the vertices of the main diagonal of coordinates X_SC_ = 225, Y_SC_ = −225, and Z_SC_ = 600 for the SS1 set, and coordinates X_SC_ = −225, Y_SC_ = 225, and Z_SC_ = 0 for the SS2 set, with respect to the satellite coordinate system (X_SC_, Y_SC_, and Z_SC_) and supported on the panels (X+ and X−) that fix the solar cells, as shown in Figure 2. The sets are located on opposite corners of the satellite, to ensure that at least one of them is illuminated by the Sun, regardless of the satellite’s attitude within the orbit.

**Figure 3:**
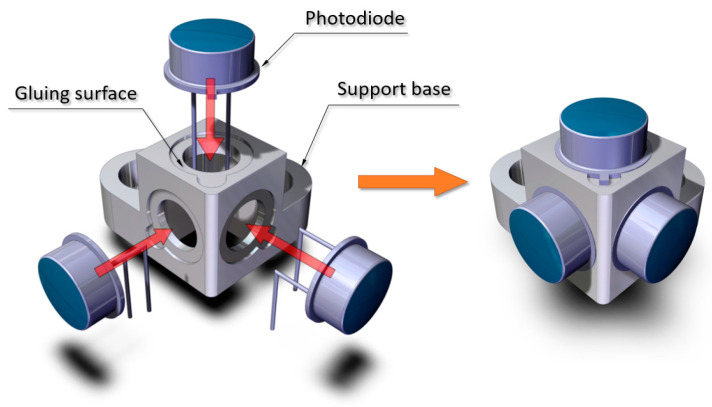
Assembly of the photodiodes on the support base.

Each set is composed of 3 OSRAM BPX 61 type photodiodes attached to a support base using two-component structural adhesive F-2216B/A (Scott Howell 3M St. Paul, MN, USA), as shown in Figure 3.

**Figure 4:**
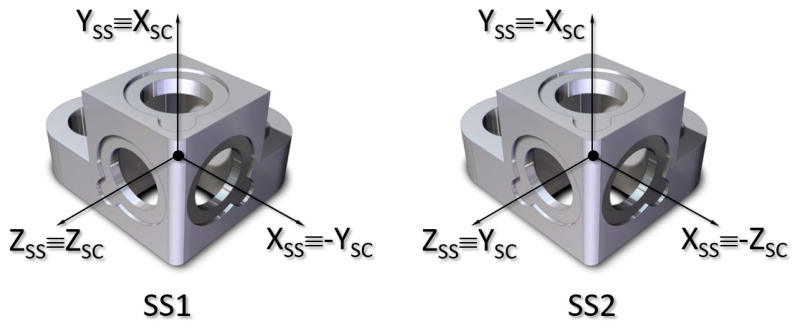
Solar sensor reference system (X_SS_, Y_SS_, and Z_SS_) and its correspondence with the satellite reference system (X_SC_, Y_SC_, and Z_SC_) for the SS1 sensor and the SS2 sensor, respectively.

The support base is designed and manufactured by IDR/UPM in Delrin^®^ 100 acetal homopolymers resin from DuPont™( DuPont Corporation Wilmington, Delaware, USA), and whose objective is to ensure the correct position of the three photodiodes in three perpendicular directions that define the sensor coordinate system (X_SS_, Y_SS_, and Z_SS_) (see Figure 4).

The support base is manufactured by milling and a dimensional verification process is carried out, on a calibrated table, of the perpendicular tolerance of each axis (∅0.01 mm) and the flatness tolerance of the support surface (0.01 mm).

**Figure 5:**
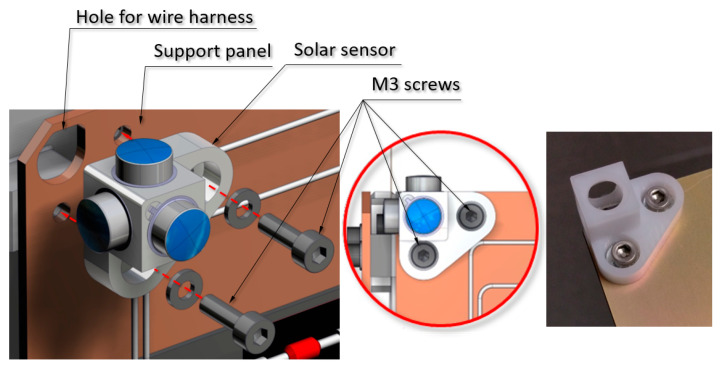
SS1 solar sensor assembly on the support panel attached using two M3 screws.

Each sensor assembly is fastened to the panels by means of two M3 screws. The position tolerance (∅0.05 mm) with respect to the side faces of the panel where the sensor is attached has been verified (see Figure 5).

### 2.2. Sun Sensor Electrical Design

The sun sensor designed and constructed for the UPMSat-2 is composed of six OSRAM BPX 61 photodiodes. They were selected as positioning systems based on photodiodes, as they have proven to offer adequate accuracy [15]. In addition, this specific model is capable of operating in quite a large temperature range (from *T* = −40 °C to *T* = 125 °C), has wide field of view, and the output current (70 nA/lx) guarantees sufficient signal-to-noise ratio.

**Figure 6:**
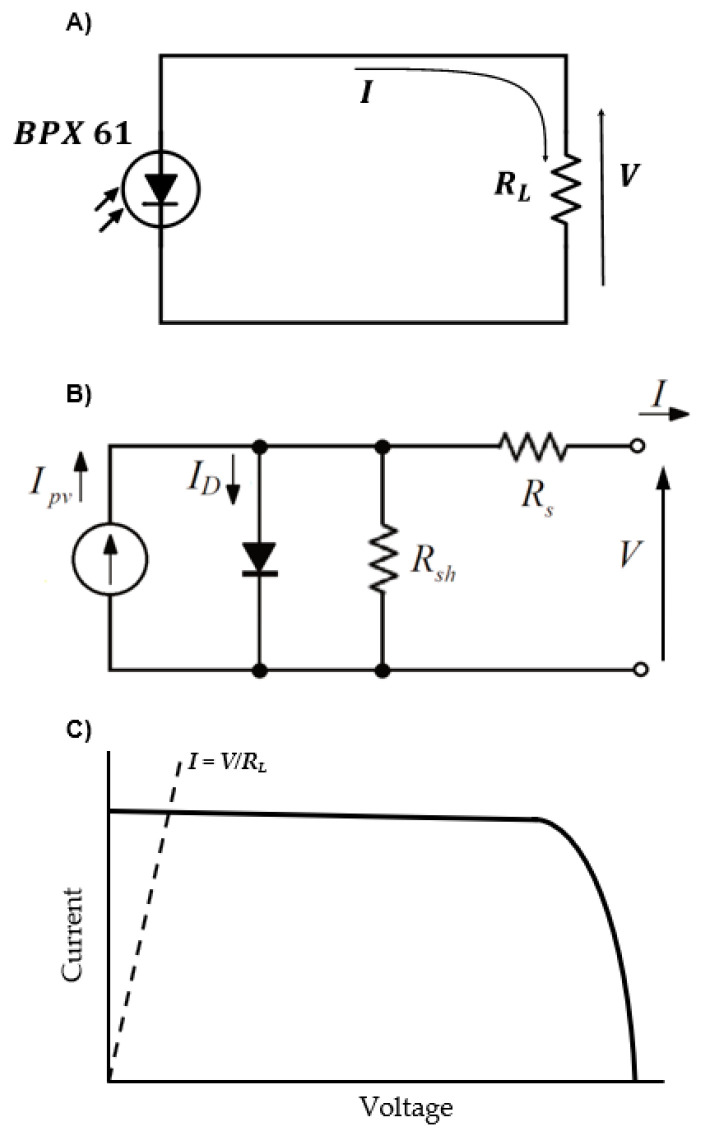
(**A**) Sketch of the electric circuit composed to measure the *I–V* curve of the photodiodes. (**B**) 1-Diode/2-Resistor equivalent electrical model used for the photodiode. (**C**) *I–V* operating curve of a photodiode.

The photodiodes are connected to a 5 V channel of the satellite’s Power Distribution Unit (PDU) and to a resistance of *R_L_* (see Figure 6A). The voltage drop across the load resistance, due to the current produced by the photodiodes, is recorded by the OBC (On Board Computer).

In order to choose the most suitable value of the resistance *R_L_*, it is necessary to know how a photodiode performs and, if possible, to model its performance, as this will also help to predict its behavior in orbit. There are different models to approximate the electrical behavior of a photodiode. Among them, the 1-Diode/2-Resistor equivalent circuit model was selected, see Figure 6B. It is basically composed of a current source connected in parallel with a diode, together with a shunt resistance *R_sh_*, and a series resistance *R_s_* [16,17,18,19]. Using the Shockley model for the diode, the output current, *I*, and the output voltage, *V*, at the terminals of the photodiode are related implicitly by the following equation:

(1) $$I=I_{pv}-I_{0}\left[\exp\left(\frac{V+IR_{s}}{aV_{T}}\right)-1\right]-\frac{V+IR_{s}}{R_{sh}},$$

where *I_pv_* is the photovoltaic current, *I*_0_ is the reverse saturation current of the diode, *a* is the ideality factor, *R_sh_* is the shunt resistor, *R_s_* is the series resistor, and *V_T_* is the thermal voltage of the diode, which is expressed in terms of the temperature, *T*, the Boltzmann’s constant, *κ*, and the electron charge *q* as:

(2) $$V_{T}=\frac{\kappa T}{q}.$$

The 1-Diode/2-Resistor equivalent circuit model defined by Equation (1) contains five parameters: *I_pv_*, *I*_0_, *a*, *R_sh_*, and *R_s_*, that need to be determined by experimental measurements, or extracted from a reduced number of characteristic points from the *I–V* curve. For a given light spectrum, the photovoltaic current *I_pv_* generated by the photodiode is proportional to the illuminance intensity. In Figure 6C a sketch of the typical *I–V* curve of the performance of a photodiode is presented. Parameters of Equation (1) must be adjusted with experimental data or those provided by the manufacturer in order to emulate the *I–V* curve. This process is detailed in Section 3.1, and real *I–V* curves obtained in tests can be seen in Figure 10.

In the event that it is desirable to obtain the maximum power of the photodiode (as is the case of solar panels), the resistance *R_L_* should be chosen so that the operating point is close to the elbow of the *I–V* curve in Figure 6C. However, the objective of the sensor is to accurately measure irradiance, and that area of the curve does not have a linear behavior with irradiance, and especially with temperature, which would complicate the measurements. Therefore, it is more desirable for the sensor to operate near the short-circuit point (*V* = 0), where the behavior is more linear. For this, the resistance *R_L_* should be small enough. Nevertheless, if the resistance is too small, the voltage generated will also be too small, making it difficult to measure and requiring greater precision. Due to this reason, it is more appropriate to have a large resistor to improve the resolution. The solution is, therefore, to choose the highest resistance that guarantees the performance within the linear zone of the *I–V* curve. In the case of the photodiodes analyzed, the resistance chosen was 32 Ω, as it met these requirements.

Finally, it must be considered that the *I–V* curve and the parameters that describe it will change depending on the operating temperature [20]. Therefore, it is desirable to include a temperature sensor within the solar sensor. In the case of the UPMSat-2 the connections were limited, so it was not possible to include this sensor in the design. However, a temperature sensor from the thermal control experiment was placed close enough to each of the solar sensors to give a good approximation of the temperature at which they would operate.

## 3. Testing Set-Up and Methodology

The fitting and testing processes described in this section were carried out by using low-cost methodology and the instrumentation available at an academic laboratory, but always maintaining the rigor and precision of the results. Two different tests were carried out: an illumination test, and an angular response test.

### 3.1. Illumination Test

The goal of the illumination test is to measure the response of the photodiodes to the light they will receive in orbit. Ideally, a AM0 spectrum light source is desirable, as this will be the spectrum the photodiodes will receive on-flight. However, if this source of light is not available, it is possible to characterize the in-flight performance of the photodiode sensors from the response to other sources of light and the information available in the manufacturer’s datasheet. Given the light source spectrum and the photodiode relative spectral response, the performance of the photodiodes can be correlated to obtain the response to any other source of irradiation, in this case the response to the AM0 spectrum.

**Figure 7:**
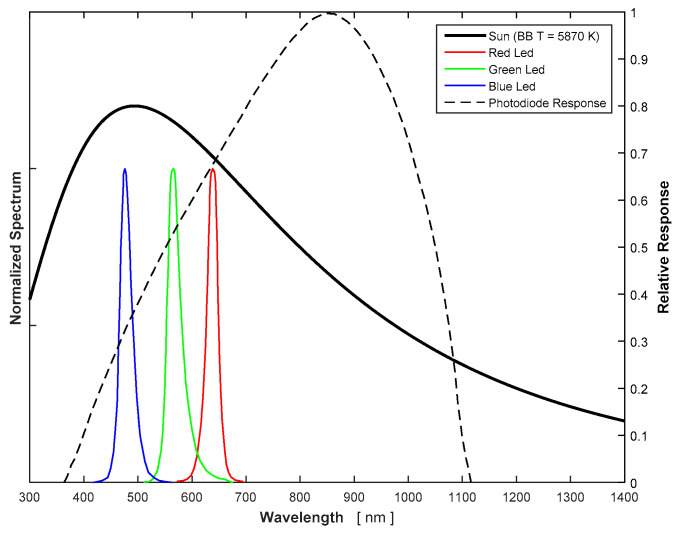
Typical spectrum of different light sources and the photodiode response.

The light sources considered were solar, halogen, incandescent, and light-emitting diode (LED) illumination. However, natural solar light was discarded to avoid uncertainties due to atmospheric absorption of certain wavelengths, and in favor of a more controlled environment in the laboratory. Among the rest of the sources, red LEDs were selected as the best option in terms of cost and due to their well-known spectrum, which is included in the manufacturer’s datasheet. Additionally, halogen was used as a secondary source of light in order to validate the results independently. The peak response of the UPMSat-2 photodiodes is in the near-infrared region of the spectrum (see Figure 7), therefore, infrared LEDs could have been a better selection. However, the available instrumentation to measure the irradiance level reaching the photodiode sensor indirectly, a CEM-DT 1308 lux meter [21], only measures in the visible light range. Red light is, therefore, a tradeoff source that can be measured with a lux meter and still provides a significant response of the photodiode.

The illumination test can be summarized as follows. The sensor is exposed to the light source with a known illuminance (calibrated in the case of the halogen light and measured with a luxmeter for the red LED). The electrical circuit (see Figure 6) is completed with a resistance *R_L_*, and the voltage *V* between its terminals is measured. The *I–V* curve of the direct polarization zone is obtained by changing the value of *R_L_* while maintaining a constant illuminance. The response with each source of light is completely characterized after repeating the process for different illuminance values.

**Figure 8:**
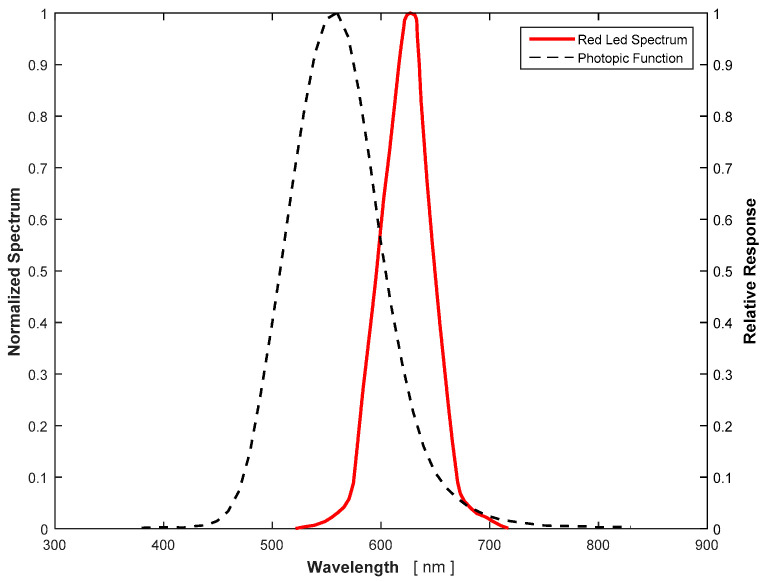
Normalized red led spectrum and photopic function.

As mentioned above, a correlation process is required to predict the response of the photodiode to any light spectrum. The first step is to calculate the spectral irradiance that reaches the photodiode from the illuminance value given by the luxmeter. The illuminance provided by the luxmeter stands for the perceived brightness by the human eye, and it is a wavelength average of the spectral irradiance to account for the sensitivity of the human eye to different wavelengths, that is, the photopic function (see Figure 8). Mathematically, it can be expressed as:

(3) $$E_{v}=C_{v}\int_{\lambda }V\left(\lambda \right)\;E_{\lambda }\left(\lambda \right)\;d\lambda ,$$

where *E_λ_* is the spectral irradiance of the light source at the lux meter sensor. The spectral sensitivity *V*(*λ*) is the built-in function of the lux meter, in this case, the photopic function. As the photopic function is a normalized curve, a constant *C_v_* = 683 lm/W is used to obtain the illuminance in SI units (lx). If no absorption of any particular wavelength between the light source and the photodiode sensor is considered, the emission spectrum of the light source is the same as the one reaching the photodiode sensor. The spectral irradiance can be then expressed as the product of the normalized irradiance *Ê_λ_*. *Ê_λ_*, the normalized irradiance of the light source, in the case of the LEDs used, is data provided by the manufacturer (see Figure 8 and [22]). *K* is then the constant that measures the magnitude of the irradiance that reaches the sensor measured in W/m^2^ nm. Therefore, the following equation can be derived:

(4) $$E_{\lambda }\left(\lambda \right)=K\;{\hat{E}}_{\lambda }\left(\lambda \right)\;.$$

Provided that the distance between the light source and the orientation of the lux meter and the photodiode sensors are the same (this can be ensured by the set-up), the constant *K* can be determined from the lux meter *E_v_* measurements as:

(5) $$K=\frac{E_{v}}{C_{v}\int_{\lambda }V\left(\lambda \right)\;{\hat{E}}_{\lambda }\left(\lambda \right)\;d\lambda }\;.$$

**Figure 9:**
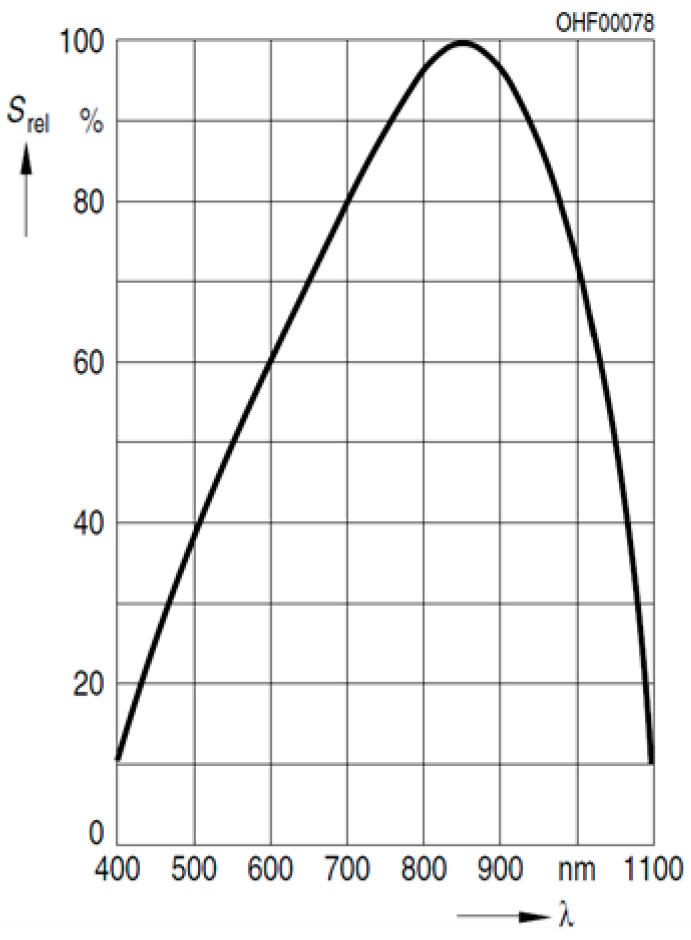
Relative Spectral Sensitivity function of the sensor *Ŝ_λ_* [21].

Then, the spectral radiance flux that reaches the sensor can be expressed as:

(6) $$\Phi_{\lambda }\left(\lambda \right)=A_{s}E_{\lambda }\left(\lambda \right)=A_{s}K{\hat{E}}_{\lambda }\left(\lambda \right),$$

where *A_s_* is the normal sensitivity area of the photodiode (this information is provided by the manufacturer, see [22]). However, not all the irradiance that reaches the photodiode is transformed into electrical power, as there is an efficiency associated with the process. The effective radiant flux Φ*_eff_* the sensor converts into current can be obtained by using the spectral radiant flux, Φ*_λ_*, and the normalized spectral sensitivity function of the sensor *Ŝ_λ_*, which is provided by the manufacturer (see Figure 9):

(7) $$\Phi_{eff}=\int_{\lambda }{\hat{S}}_{\lambda }\left(\lambda \right)\;\Phi_{\lambda }\left(\lambda \right)d\lambda =A_{s}\int_{\lambda }{\hat{S}}_{\lambda }\left(\lambda \right)\;E_{\lambda }\left(\lambda \right)d\lambda .$$

The next step in this process is to correlate the effective radiant power in the photodiode sensor with a measurable response of the photodiode at its terminals, that is, voltage and current. The manufacturer of the photodiode provides the radiant sensitive area of the sensor, *A_s_*, the normalized sensitivity function, *Ŝ_λ_*, and the sensitivity constant, *S_λ_* (with dimensions of A/W). These variables are required to translate the spectral irradiance, *E_λ_*, in terms of generated current, *I_pv_*:

(8) $$I_{pv}=S_{\lambda }\Phi_{eff}\;=S_{\lambda }A_{s}\;\int_{\lambda }{\hat{S}}_{\lambda }\left(\lambda \right)\;E_{\lambda }\left(\lambda \right)d\lambda \;=P\;\int_{\lambda }{\hat{S}}_{\lambda }\left(\lambda \right)\;E_{\lambda }\left(\lambda \right)d\lambda \;=PE_{eff},$$

where *P* is a new parameter, defined as the product of *S_λ_*, the spectral sensitivity of the sensor, and *A_s_*, which is also an intrinsic characteristic of each photodiode. The constant *P* can be seen as the conversion efficiency between the effective irradiance *E_eff_* and the generated current. The manufacturer’s values of *Ŝ_λ_*, *A_s_*, and *S_λ_* allow us to directly obtain the response of the photodiode at any spectrum, and specifically at AM0. However, these are typical values, not the photodiode exact ones. In addition to the parameters *I*_0_, *a*, *R_sh_*, and *R_s_*, the goal of the fitting process is to determine a more representative value of the product *S_λ_A_s_*, that is, the parameter, *P*. Ideally, the relative spectral sensitivity *Ŝ_λ_* should also be characterized but, regrettably, no equipment to perform that calibration was available. Instead, the typical one provided by the manufacturer was used. In the following section, it is concluded that using the manufacturer’s *Ŝ_λ_* (among other sources of errors), yields to some discrepancies in the response to different light spectrums. However, the values for the constant *P* obtained by following the procedure described in the present work seem more reliable than the ones provided by the manufacturer. Therefore, a full fitting process would consist of the extraction of five parameters: *P*, *I*_0_, *a*, *R_sh_*, and *R_s_*, from the measured data. In this case the parameter *I_ph_*, that can be obtained from the fitting, is replaced with *P* that, for a given illuminance, can be obtained from the first one using the following equation derived from (8):

(9) $$P=\frac{I_{pv}}{K\int_{\lambda }{\hat{S}}_{\lambda }\left(\lambda \right)\;{\hat{E}}_{\lambda }\left(\lambda \right)d\lambda }.$$

The advantage of using *P* is that this parameter is independent on the illuminance, while *I_ph_* changes with it. With enough measurements, the fitting process can be seen as an optimization problem with five degrees of freedom. The objective function to minimize is defined as the Root Mean Square Error (RMSE) between the model and the experimental data:

(10) $$\mathrm{RMSE}=\sqrt{\frac{1}{n}{\sum_{i=1}^{n}\left(I_{i,}{}_{exp}-I_{i,}{}_{model}\right)}^{2}},$$

where *n* is the number of measurements, *I_i,exp_* is the measured current. and *I_i,model_* is the current predicted by the model. Different optimization algorithms can be used to obtain the parameters of the model. Non-linear least squared solvers and quasi-Newton methods are suitable for this task. Once the parameters are obtained, it is possible to predict the response of the photodiode to any light spectrum and for any resistance load between the terminals.

Two different testing sets were carried out at two illuminance conditions (88,500 and 47,000 lx), each one composed by two different measurements of the output voltage, *V*, varying the resistor value, *R_L_*, of the load connected to the photodiodes’ terminals (see in Table 1 the data corresponding to SS1-X+ photodiode). The output current is derived from the well-known Ohm law:

(11) $$V=R_{L}I$$

**Figure 10:**
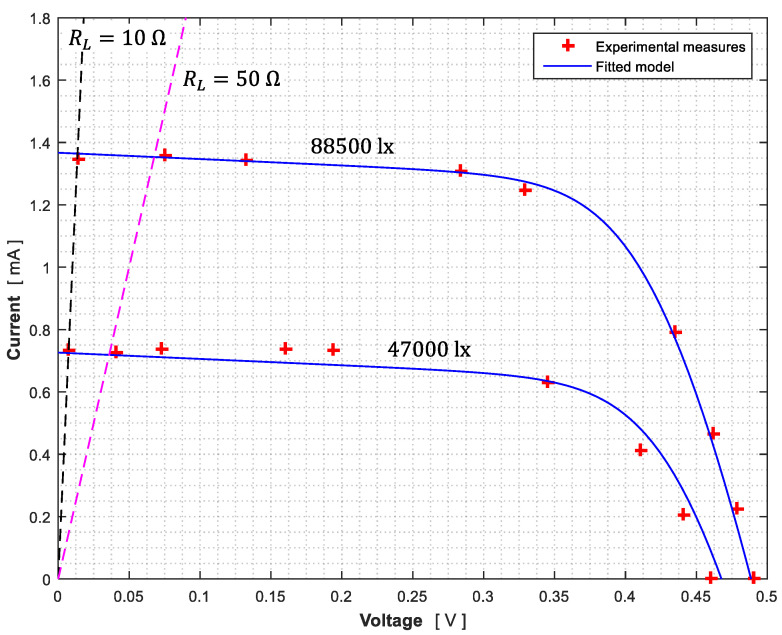
Results of the of the illuminaton testing campaign. Photodiode SS1-X+ output current related to the output voltage (see also Table 2). The fittings of the 1-D/2-R equivalen circuit model to the data have been included in the graph. The dashed lines representing *R_L_* = 10 Ω and *R_L_* = 50 Ω have been also incuded in the graph.

For each illuminance condition of Table 1, the mean values of both sets of measurements, and a solid line representing the fitted model corresponding to Equation (1), have been plotted in Figure 10. The parameters of these fittings are included in Table 2. As seen in the figure, the fitted model seems to reproduce approximately the photodiode response. Additionally, the dotted lines represent the possible working region of the photodiode in which the value of the resistor is kept between *R_L_* = 10 Ω and *R_L_* = 50 Ω.

Once the *I–V* curve is obtained, it is possible to select the load resistance *R_L_* that is connected to the terminal of the photodiodes. In the UPMSat-2 the voltage across the load resistance is measured through the Analog to Digital Converter (ADC) of the On-Board Computer (OBC). Ideally, a bigger value of the load resistance *R_L_* is desirable to increase the resolution, that is, to maximize the voltage response to the effective irradiance, $dV/dE_{eff}$. However, especially at low temperatures the response curves $dV/dE_{eff}$ become non-constant (non-linear response) for high *R_L_* values. It was estimated that a value of the load resistance lower than 50 Ω is required to keep the photodiode in the linear response area for all the temperature ranges estimated for the UPMSat-2 mission.

The resulting fitted model was also evaluated with the other light source. For this evaluation, the photodiode was exposed to a halogen source of light whose irradiance spectrum was previously measured. The lamp was characterized at the facilities of CIEMAT (*Centro de Investigaciones Energéticas, Medioambientales y Tecnológicas*), which is the public research body devoted to energy and environment and the technologies related to them in Spain [23]. In this case, the lux meter was not required, as the spectral irradiance of the light source, *E_λ_*, was known, and the effective irradiance could be directly calculated with Equation (8). After measuring the response of the photodiode at different resistor loads, the correlation carried out for the red LEDs was repeated in order to derive the parameters of the fitted model. The results showed almost the same values (with differences around 3%, e.g., *P* = 5.39·10^−6^ Am^2^/W in the checking results instead of *P* = 5.56·10^−6^ Am^2^/W). Considering that no dedicated optical equipment was used in the illumination tests, and that all the spectral properties were collected from the manufacturers’ datasheets, the discrepancy seems not to be large. Finally, it should also be pointed out that the typical value provided by the manufacturer (*P* = 4.35·10^−6^ Am^2^/W) did not properly represent the conversion efficiency.

Once the red LED results were validated with the halogen measurements, the behavior of all the photodiodes to the AM0 spectrum was estimated. However, a complete characterization such as the one shown in Figure 10 for all the remaining sensors (SS1-Y−, SS1-Z+, SS2-X−, SS2-Y+, and SS2-Z−) was not needed, as the operation voltage was already determined. Instead, a simpler fitting was carried out, measuring only the voltage across a constant load (31.4 Ω) similar to the flight one (32 Ω). As stated above, near the short circuit region, *R_L_* < 50 Ω, the response $dI/dE_{eff}$ is almost constant. Therefore, the response in current to a change in the illumination conditions is linear. By knowing the resistance, and measuring the voltage across the load at different illuminance values with the red LEDs, it is possible to estimate the value of the slope $dI/dE_{v}$ quite accurately, and then it is possible to derive the proper value of $dI/dE_{eff}$, which gives the response of the sensor for any light spectrum.

**Figure 11:**
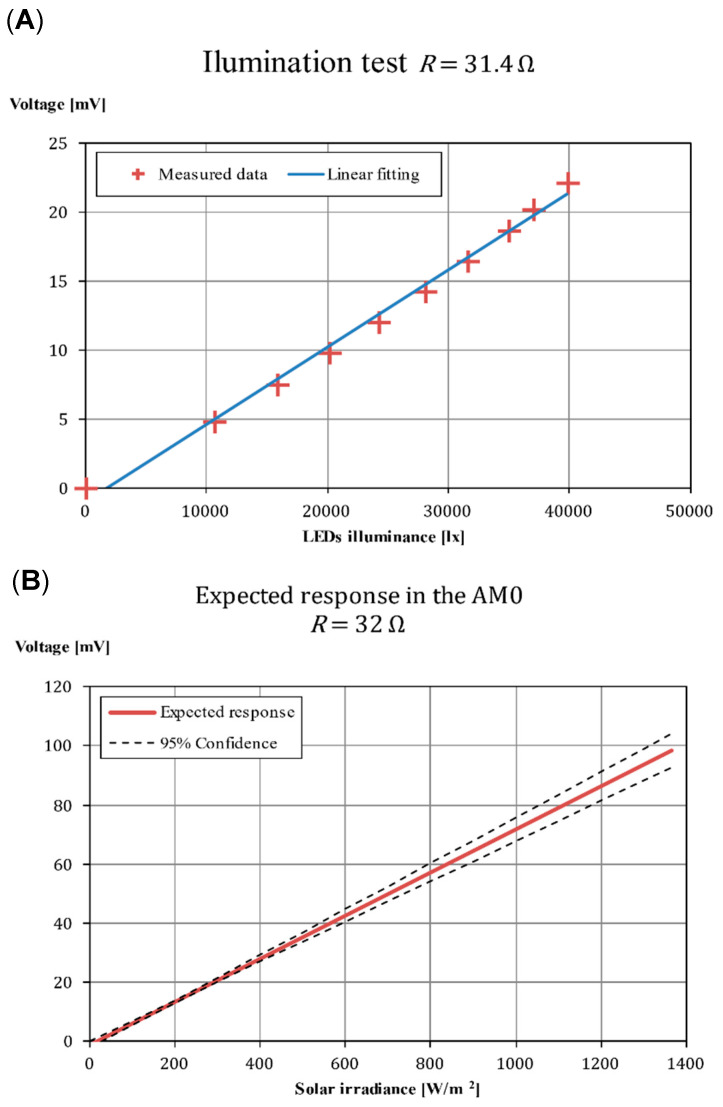
(**A**) Fitting of the SS1-X+ sensor. (**B**) Expected response of SS1-X+ sensor to AM0 spectrum irradiance.

In Figure 11, the results obtained from the LED illumination test to the SS1 flight photodiode are shown as an example, with the rest of the photodiodes showing similar behavior. As it can be appreciated, the results are obtained for a load of 31.4 Ω, which was close to the final value selected for the flight resistance (32 Ω). The slope of $dV_{\exp}/dE_{v}^{\mathrm{LED}}$ is determined through a linear fitting (see Table 3). Then, taking in account that $dI/dE_{eff}$ is a constant of the photodiode, and relating the *E_eff_* of the LED and the AM0, the variation of the current, *I*, with the solar radiation can be calculated as:

(12) $$\frac{dI}{dE^{\mathrm{AM}0}}=\frac{dI}{dE_{v}^{\mathrm{LED}}}\frac{C_{v}\int_{\lambda }V\left(\lambda \right){\hat{E}}_{\mathrm{LED}}\left(\lambda \right)d\lambda \;\int_{\lambda }{\hat{S}}_{\lambda }\left(\lambda \right)\;{\hat{E}}_{\mathrm{AM}0}\left(\lambda \right)d\lambda }{\int_{\lambda }{\hat{S}}_{\lambda }\left(\lambda \right)\;{\hat{E}}_{\mathrm{LED}}\left(\lambda \right)d\lambda },$$

where:

(13) $$\begin{array}{l} \int_{\lambda }V\left(\lambda \right){\hat{E}}_{\mathrm{LED}}\left(\lambda \right)d\lambda \;=6.97\\ \int_{\lambda }{\hat{S}}_{\lambda }\left(\lambda \right)\;{\hat{E}}_{\mathrm{LED}}\left(\lambda \right)d\lambda =15.5\\ \int_{\lambda }{\hat{S}}_{\lambda }\left(\lambda \right)\;{\hat{E}}_{\mathrm{AM}0}\left(\lambda \right)d\lambda =0.416 \end{array}$$

Finally, taking into account that the flight resistance is *R*_flight_, the relation between the experimental voltage, *V*_exp_, and the flight voltage, *V*_flight_, for the same current is:

(14) $$V_{\mathrm{flight}}=V_{\exp}\frac{R_{\mathrm{flight}}}{R_{\exp}}.$$

Therefore, the value of $dV_{\mathrm{flight}}/dE^{\mathrm{AM}0}$ (see Table 3) can be calculated with:

(15) $$\frac{dV_{\mathrm{flight}}}{dE^{\mathrm{AM}0}}=\frac{dV_{\exp}}{dE_{v}^{\mathrm{LED}}}\frac{R_{\mathrm{flight}}}{R_{\exp}}\frac{C_{v}\int_{\lambda }V\left(\lambda \right){\hat{E}}_{\mathrm{LED}}\left(\lambda \right)d\lambda \;\int_{\lambda }{\hat{S}}_{\lambda }\left(\lambda \right)\;{\hat{E}}_{\mathrm{AM}0}\left(\lambda \right)d\lambda }{\int_{\lambda }{\hat{S}}_{\lambda }\left(\lambda \right)\;{\hat{E}}_{\mathrm{LED}}\left(\lambda \right)d\lambda }.$$

No variation of the temperature was considered in the present work as the instrument does not include an internal temperature sensor. The temperature of the photodiodes was *T* = 22 °C during the illumination testing campaign, and *T* = 24 °C during the angular testing. Quite large changes in this variable are expected due to the day–night transitions [24] and these changes can be taken into account when post-processing data from the UPMSat-2 mission. Nevertheless, for the UPMSat-2 operating temperature range, the response of the photodiodes is expected to change with respect to the predictions at room temperature by a maximum of a 5%, this figure being in the same order of magnitude as the error obtained between the LED illumination and the calibrated halogen lamp. Therefore, this error was assumed as acceptable in this testing campaign (see also Appendix A). The estimated response has therefore also plotted together with the 95% confidence interval calculated from the linear regression (dashed lines in the right graph of Figure 11). If greater precision is required, it is recommended to include a temperature sensor in the instrument and extend the test campaign at different operating temperatures.

### 3.2. Angular Response

In the directional test, a single resistance (31.4 Ω) and illumination is used and the angle that the sensor makes with the direction of the light source is varied. The goal of the angular testing campaign is to determine the response of the photodiode when the angle between the normal of the sensor and the light source is changed. For this test, a source of light with parallel rays is needed. The source was the halogen lamp, and several screens were used, to ensure that the incident light is properly aligned with the sensor main direction.

**Figure 12:**
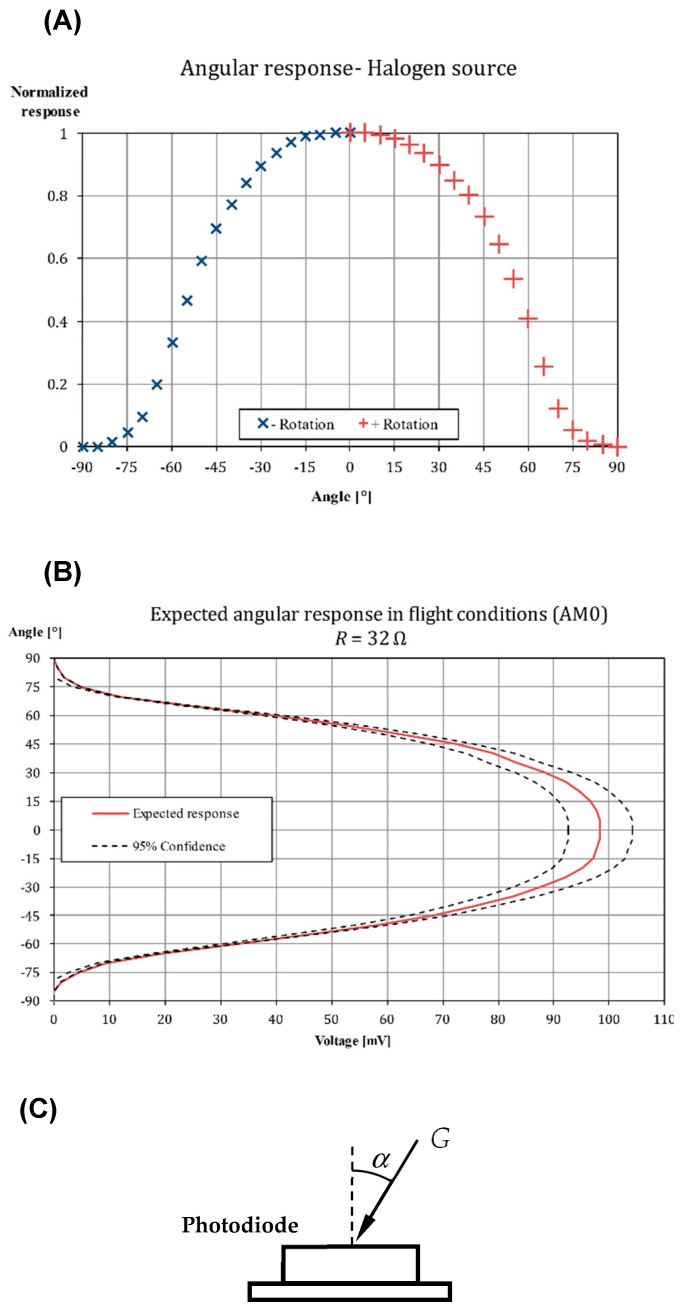
(**A**) Response of the photodiode SS1-X+ directional test. (**B**) Expected angular response of the photodiode SS1-X+ in AM0 spectrum. (**C**) Sketch of the photodiode under the irradiance, *G*, at angle *α*.

Voltage from the photodiodes under different directions of incidence of the light have been measured and normalized with the maximum one. In Figure 12, top graph, this measurement is presented for SS1 flight photodiode, the rest of them showing a similar behavior (see Table 4). With the normalized angular response of the photodiodes, and knowing its voltage response depending on the irradiance in the AM0 spectrum (Figure 11, bottom graph), the angular response in flight conditions is obtained (Figure 12, bottom graph). For this calculation, it must be considered that the irradiance received will be the one corresponding to AM0 for a certain *α*. This curve determines the angle between the Sun and the normal to the sensor *α* (see Figure 12 final sketch) as a function of the voltage readings of the On-Board Computer. As can be seen in this latter graph, when the Sun is perpendicular to the sensor (that is, for small *α*), taking into account the error introduced in Figure 11 due to the temperature, it is not possible to determine the angle with precision. However, the uncertainty is greatly reduced as the angle increases, the 95% confidence interval being only a few degrees in size for angles between 45° and 75°. For the UPMSat-2 this is not problematic, as the sun sensors are a secondary payload and the attitude is primarily determined through magnetometers. Additionally, the information from the sun sensors can be filtered (*i.e.*, Kalman filter) when the satellite is in normal mode (spin stabilized), to increase the accuracy of the calculated direction of the Sun irradiance within the brackets (0°, 45°) and (75°, 90°). This “in orbit” improvement of the sun sensor performance is one of the different experiments programmed in the UPMSat-2 mission.

In order to easily convert the voltage measured in the angle of incidence of the Sun, the performance of each photodiode, that is, the angle, *α*, of the Sun direction in relation to the normal direction to its surface, is modelled with a 7th-degree polynomial:

(16) $$\alpha =a_{0}+{\sum_{n=1}^{7}a_{n}\left(\frac{V}{V_{\max}}\right)}^{n},$$

where *V* is the voltage measured by the sensor, and *V*_max_ is the maximum value when the Sun direction is perpendicular to the sensor’s surface (see Table 4).

The 7th degree polynomial function, selected as the relative response of the photodiodes used in the UPMSat-2, showed a significant discrepancy between the measured values and the cosine law for angles larger than 45°. To maintain the highest possible accuracy, the experimental data was fitted to different possible transfer functions, the 7th degree polynomial expression being the lowest degree polynomial function that showed good results and consistency between the different photodiodes tested.

### 3.3. Sun Direction

Finally, all voltage measurements from the photodiodes from both sun sensor platforms of the satellite, SS1, and SS2 (see Section 2), are required together to obtain the three components of the direction of the Sun in relation to the satellite. Bearing in mind that the sensors of one platform are oriented to the three principal direction axes of the satellite (*x*, *y*, and *z*), it is possible to derive the three components of the Sun direction (*s_x_*, *s_y_*, and *s_z_*, in the satellite reference coordinate system), as:

(17) $$\left(s_{x},s_{y},s_{z}\right)=\left(\pm \cos\left(\alpha_{x}\right),\pm \cos\left(\alpha_{y}\right),\pm \cos\left(\alpha_{z}\right)\right),$$

where the sign of each component depends on which platform is illuminated by the Sun. Therefore, it can be said that the values of each coordinate depend on the measurements of a pair of photodiodes (e.g., +*x* and −*x* photodiodes define *x*-axis coordinates).

## 4. On-Orbit Sensors’ Performance

The UPMSat-2 is placed in a 525 km altitude 10:30 Sun-synchronous orbit, its *z*-axis being perpendicular to the plane of the orbit and with approximately *ω* = 0.02 rad/s rotation rate around this axis. The ADCS of the UPMSat-2 is purely magnetic. It is based on a modification of the B-dot control law that allows control of the satellite’s rotation rate and, at the same time, orients the spin axis perpendicular to the orbit. Placing the satellite perpendicular to the orbit ensures good illumination of the lateral solar panels and an adequate orientation of the antennas for communications. In addition, the rotation speed can be adjusted to improve the thermal control of the satellite and prevent the panels from overheating. One of the advantages of this control law is also its simplicity, as it does not need to determine the attitude of the satellite as it only needs the magnetic field direction. Therefore, the ADCS control system of UPMSat-2 does not need attitude determination to operate, only magnetic field direction. Data measured in orbit on the UPMSat-2 mission are downloaded from the satellite and processed in order to obtain the orientation.

### 4.1. Results

The satellite measures the output voltage of each photodiode. In order to obtain the Sun direction, it is necessary to estimate the maximum voltage response of each photodiode taking into account the environmental conditions. The main factors affecting the maximum output current of a photodiode are the sensor temperature and the irradiance reaching the photodiode. The maximum expected output voltage of the photodiode can be obtained with:

(18) $$V_{max}=M\cdot G\cdot e^{\phi (T_{ss}-T_{ref})}+n,$$

where *M* and *n* are the fitted coefficients obtained from the solar sensor illuminance test (see Table 3), *ϕ* is the current temperature coefficient extracted from the photodiode datasheet, *T_ss_* is the temperature of the photodiode, while *T_ref_* is the reference temperature of the photodiode during the testing process (*T_ref_* = 22 °C), and *G* is the solar constant (*G* = 1360 W/m^2^). Although the solar irradiance value changes thought the year, its variation is not greater than 5%, making no significant differences in the results obtained.

The Sun is not the only source of light that reaches the photodiodes. Other (undesired) sources should be considered as well, the main one being the Earth’s albedo radiation, whose contribution can be especially high for satellites that operate in a polar orbit (mainly as ice and snow have a high reflectance coefficient). Considering that the photodiodes present a linear behaviour in the proximities of the working point, the albedo contribution can be estimated and corrected from:

(19) $$V=V_{s}+V_{\alpha },$$

where:

(20) $$V_{\alpha }=M\cdot G_{\alpha }\cdot e^{\phi (T_{ss}-T_{ref})}+n.$$

The Earth’s albedo contribution to the overall voltage, *V_α_*, can be obtained using a similar procedure to the one used to obtain the maximum expected voltage due to solar radiation. In this case, it is necessary to calculate the total irradiance from Earth that is reaching the photodiodes, *G_α_*, using an albedo model. The albedo model implemented in this work is mainly based on O’Keefe and Schaub’s work [25]. In this model, the Earth is discretised in differential areas, Δ*A*. Each differential area is associated to the coordinates of its centroid, **r***_A_*, and its average albedo value is *a_i_*. These *a_i_* values can be obtained from experimental data or calculated using a model. In this work, the average albedo values were obtained using the albedo model recommended by the ECSS standard [26]. Taking into account the Sun and satellite positions in relation to the Earth, the total amount of radiation that reaches the satellite can be computed with:

(21) $$G_{\alpha }=-\frac{G}{\pi }\sum_{i=1}^{n}\frac{c_{i}a_{i}}{{\left\|r_{A_{i}B}\right\|}^{2}}\left(\frac{n_{A_{i}}^{T}s_{⊕}}{\left\|n_{A_{i}}\right\|\left\|s_{⊕}\right\|}\right)\left(\frac{n_{A_{i}}^{T}r_{A_{i}B}}{\left\|n_{A_{i}}\right\|\left\|r_{A_{i}B}\right\|}\right)\left(\frac{n_{ss}^{T}r_{A_{i}B}}{\left\|n_{ss}\right\|\left\|r_{A_{i}B}\right\|}\right)\Delta A_{i},$$

where $r_{A_{i}B}$ is the vector from the centroid of Δ*A_i_* to the satellite, $s_{⊕}$ is the unit direction from Earth to the Sun, $n_{A_{i}}$ is the unit normal direction of Δ*A_i_*, $n_{\mathrm{ss}}$ is the normal direction of the photodiode, and *c_i_* is a coefficient whose value is 1 if a series of conditions are met and 0 in the other cases:

(22) $$c_{i}=\left\{\begin{array}{l} 1\;\mathrm{if}\;\;\left(n_{A_{i}}^{T}s_{⊕}>0\right)\;\wedge \;\left(n_{A_{i}}^{T}r_{A_{i}B}>0\;\right)\;\wedge \;\left(n_{ss}^{T}r_{A_{i}B}<0\right)\\ 0\;\mathrm{else} \end{array}\right\}.$$

One of the problems when estimating the albedo noise in the sun sensors is that the attitude of the vehicle must be known. This point could be problematic for satellites whose attitude is not constrained.

**Figure 13:**
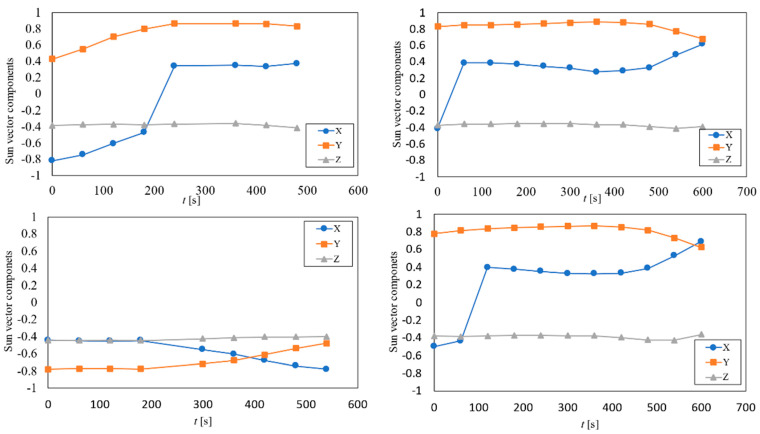
Calculated sun vector components for the illuminated accesses of the first three days of mission.

**Figure 14:**
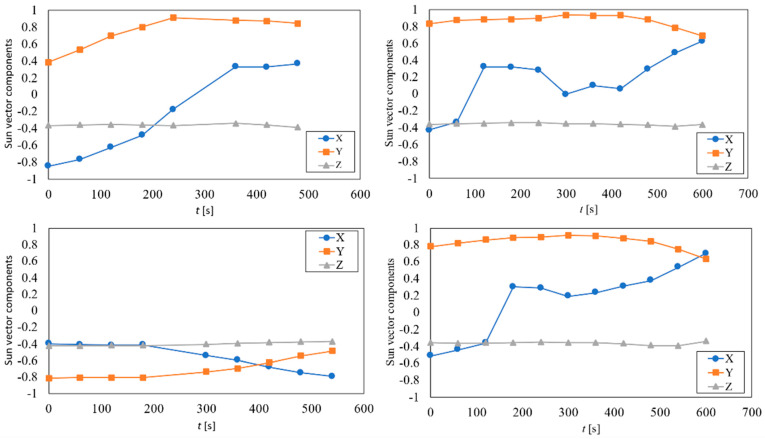
Calculated and filtered sun vector components for the illuminated accesses of the first three days of mission.

The results from UPMSat-2 data within the first three days of the mission (4th to 6th September 2020) are included in Figure 13. In this figure, the calculated Sun vector for four different accesses is represented. In each graph, the evolution of the different components of the measured sun vector in relation to the access time are shown. The results obtained show that the performance of the solar sensors is poor when the incidence angle is grater that 65°. Concerning the results obtained from the *z*-axis, a constant orientation of the satellite in relation to the Sun is shown, the angle of that axis being α_z_ ∈ (110°, 117°) based on measurements from the sun sensors. It should be emphasized that considering the error foreseen by the UPMSat-2 ADCS with regard to the perpendicularity of its *z*-axis in relation to the orbit’s plane, results are in quite good agreement with the theoretical average value within a year, α_z_ = 112.5°. However, the angular velocity of the spacecraft is left unknown, as the measurement rate is not fast enough to infer the angular velocity. In Figure 14, the results obtained if albedo noise is removed from the measurements are included. The contribution of the Earth albedo radiation is relatively small compared to the solar radiation. Nevertheless, the inclusion of this effect enhances the result obtained when the Sun falls at large angles (relative to the sensor normal direction) as can be seen when both figures are compared.

### 4.2. Validation of the Results

The Sun direction coordinates, based on both the sun sensors and the magnetometers of the UPMSat-2, were compared to analyze the results of the performance of the solar sensor and determine if the measures make sense with the expected attitude. The Sun direction in relation to the UPMSat-2, based on the magnetometers, can be derived from:

∘ The position of the satellite obtained by orbit propagation from the last TLE data;
∘ The position of the Sun in Earth-Centered Inertial (ECI) coordinates system;
∘ The magnetic field at the position of the satellite, calculated with the International Geomagnetic Reference Field (IGRF) standard;
∘ The magnetic field measured by the UPMSat-2 magnetometers.

One final hypothesis must be assumed in order to calculate the attitude of the satellite from the above data: the *z*-axis of the satellite should be reasonably perpendicular to the orbit’s plane, as stated by the control law [5,27].

**Figure 15:**
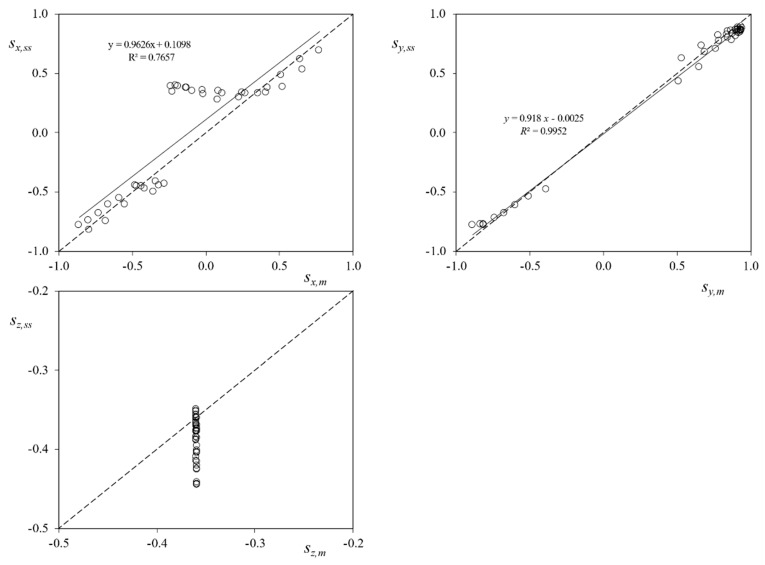
Sun direction coordinates with regard to the UPMSat-2, reference system. Coordinates based on the sun sensors system (*s_x,ss_*, *s_y,ss_*, and *s_z,ss_*) vs. coordinates based on the magnetometers of the satellite’s ADCS (*s_x,m_*, *s_y,m_*, and *s_z,m_*).

**Figure 16:**
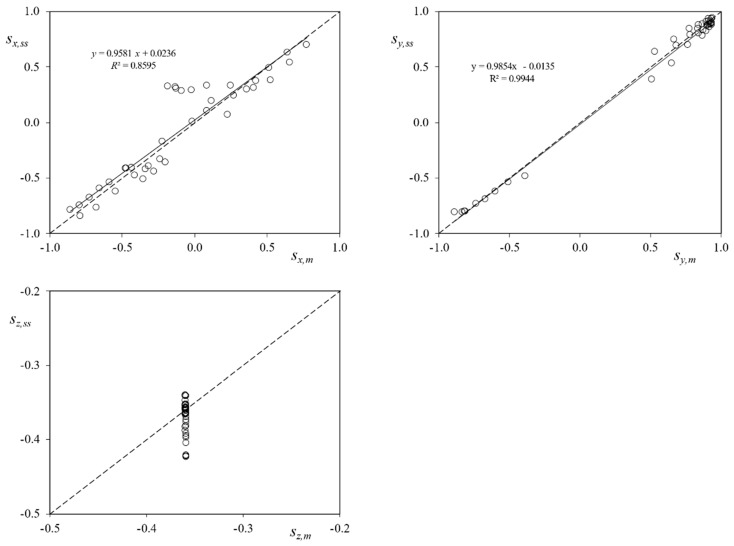
Albedo filtered sun direction coordinates with regard to the UPMSat-2 reference system. Coordinates based on the sun sensors system (*s_x,ss_*, *s_y,ss_*, and *s_z,ss_*) vs. coordinates based on the magnetometers of the satellite’s ADCS (*s_x,m_*, *s_y,m_*, and *s_z,m_*).

The results showing the validation and performance of the sun sensors are presented in Figure 15. In this figure, the coordinates of the Sun direction based on the attitude measured by the UPMSat-2 magnetometers (*s_x,m_*, *s_y,m_*, and *s_z,m_*), are plotted vs. the ones measured by the sun sensors (*s_x,ss_*, *s_y,ss_*, and *s_z,ss_*). The results in relation to each coordinate are directly compared in the graphs from the figure. Additionally, the hypothetical perfect correlation between both sets of coordinates has been included in the graphs as a dotted line. In Figure 16 the same results are presented, removing the albedo contribution.

A larger error is observed regarding the *z*-axis. This is caused by the method used to derive the satellite attitude from the magnetometer data, and the performance of the photodiodes. The attitude calculation usually requires two independent measurements. Nevertheless, the attitude could be calculated from a single measurement if there is a constraint on the attitude of the vehicle. In the present case, a 10:30 heliosynchronous orbit was considered, which imply a 67.5° between the Sun direction and the *z*-axis. However, the satellite is not perfectly aligned with the normal direction of the orbital plane and pointing errors up to 5 deg can be expected. Additionally, from Figure 12 A, it can be observed that that a small variation in the relative response produces a significant change in the calculated angle at that large and low Sun direction angles than at intermediate ones (*α* ∈ (45°, 60°)).

The results regarding the *x*-axis coordinate show a correlation with a coefficient of determination *R*^2^ = 0.7657. As expected, the data regarding directions tending to *α*_x_ = 90° or *α*_x_ = –90° reflect a lower correlation level (the lack of accuracy of the photodiodes for Sun direction angles, *α*, larger than 75° was described in the previous section). If the albedo contribution is removed, results enhance, obtaining a trend line closer to the hypothetical perfect correlation. Moreover, the coefficient of determination also increases (*R*^2^ = 0.8595).

**Figure 17:**
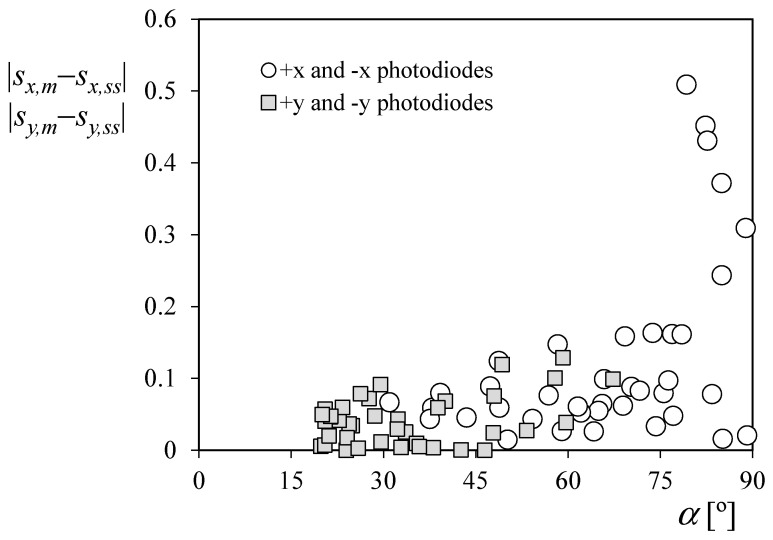
Differences between *x*-axis and *y*-axis coordinate values of the Sun direction based on the sun sensors system (*s_x,ss_*, *s_y,ss_*, and *s_z,ss_*) and on the magnetometers of the satellite’s ADCS (*s_x,m_*, *s_y,m_*, and *s_z,m_*) vs. the computed angle with regard to the corresponding illuminated photodiode using magnetometer data, *α*.

In Figure 17, the error between the *x*-axis coordinate values |*s_x,m_ − s_x,ss_*| is plotted vs. the calculated direction of the sun using the magnetometer data, *α* (see Figure 12) with respect to the active sensor of the pair related to this axis (the one corresponding to +*x* or the one corresponding to −*x*, depending on which one is illuminated by the Sun). In the graph of the figure, the larger differences for *α* > 75° can be clearly appreciated. Furthermore, if the points corresponding to these angles are left aside, the correlation between the *x*-axis coordinate values from the two different sets improves, with a coefficient of determination *R*^2^ = 0.9809.

With regard to the *y*-axis coordinate values, the correlation between the two data sets is larger, with a coefficient of determination *R*^2^ = 0.9952. This is explained as the expected Sun direction with respect to the photodiodes (+*y* or −*y*) in the analyzed points has values lower than *α* = 75°, see Figure 17. Additionally, it can be observed that, for lower values of the Sun direction, *α* → 0°, the error between coordinates seems to increase, in accordance with the results obtained in the previous section. 

Finally, the results reveal a constant orientation of the satellite *z*-axis with regard to the Sun, the angle with regard to that axis being *α*_z_ ∈ (110°, 111°) if based on measurements from the magnetometers, and *α*_z_ ∈ (111°, 117°) if based on measurements from the sun sensors, which can be improved if albedo noise is removed obtaining an angle *α*_z_ ∈ (110°, 115°). These values are in good agreement to the expected performance of the UPMSat-2 ADCS.

## 5. Conclusions

In the present work, the design, ground testing, and on-orbit performance of the UPMSat-2 sun sensor is described. This sensor was developed and built at reduced cost with COTS components. The response curve of the photodiodes was also obtained with a simple and low-cost testing process. The most relevant conclusions of this work are:

- The proposed methodology allows the user to determine the expected performance of the photodiodes in the direct polarization zone for any light spectrum without specific material;
- The results of the fitting process indicate a good performance of the photodiodes that compose the sun sensors system for Sun directions within the bracket α ∈ (45°, 75°). Accuracy could be increased by including a thermal sensor in the instrument and characterizing the response for different temperatures;
- The estimation and filtration of the Earth albedo contribution to sun sensors prove to enhance the results obtained being particularly important for angles > 75°;
- Experimental on-flight data have been obtained from the UPMSat-2 first three days of the mission (4th to 6th September 2020). The correlation between the observed coordinates of the Sun direction obtained with the sun sensors and the ones derived from the ADCS magnetometers measurements and orbit parameters indicate high reliability of the solar sensor for angles α < 75°, with poorer performance if this angle tends to α = 0°.

Promising results have been achieved, despite the sensor being developed with a very reduced budget. This kind of sensor seems very suitable for low-cost nanosatellites and microsatellites, where photodiodes are used as a backup instrumentation to determine the satellite’s attitude, especially for educational satellites such as the UPMSat-2.

## Figures and Tables

**Table 1:** Experimental measurements of the *I*–*V* curve of the photodiode SS1-X+.

*R_L_* (Ω)	Illuminance: 88,500 lx		Illuminance: 47,000 lx	
	Test 1 (mV)	Test 2 (mV)	Test 1 (mV)	Test 2 (mV)
10.0	13.2	13.7	7.4	7.3
55.6	74.2	76.6	40	40.8
98.8	132.5	132.5	73.5	72.1
216.8	280.9	286.7	159.2	160.4
264.0	329.5	328.3	192.9	193.7
548.2	434	435	343.2	347.3
994.2	462	461	411	410
2148	477	480	441	441
∞	492	488	459	461

**Table 2:** Value of the model parameters (see Equations (1) and (10)), fitted to the testing data of the photodiode SS1-X+ Figure 10.

Parameter	Fitted Value
*P* (Am^2^/W)	5.56·10^−6^
*I*_0_ (A)	1·10^−10^
*a*	1.1754
*R_s_* (Ω)	34.01
*R_sh_* (Ω)	4902

**Table 3:** Coefficients of the slope, *m*, and offset, *n*, obtained from the linear fittings to the expected response of the SS1-X+ photodiode under LED and AM0 irradiance (see also Figure 11).

Irradiance	*M*	*n*
LED	$dV_{\exp}/dE_{v}^{\mathrm{LED}}=\;5.59\cdot 10^{-4}$ mV/lx	−0.992 mV
AM0	$dV_{\mathrm{flight}}/dE^{\mathrm{AM}0}=\;7.27\cdot 10^{-2}$ mV/(W/m^2^)	−1.01 mV

**Table 4:** Coefficients of Equation (16), used for modeling the angle, *α*, of the Sun irradiance in relation to each one of the UPMSat-2 photodiodes from the SS1 and SS2 sun sensors.

Sun Sensor	*a*_0_	*a*_1_	*a*_2_	*a*_3_	*a*_4_	*a*_5_	*a*_6_	*a*_7_
SS1-X+	1.663	–10.07	91.45	–427.5	1066	–1449	1011	–283.5
SS1-Y−	1.712	–14.01	133.5	–625.7	1547	–2072	1420	–390.6
SS1-Z+	1.679	–11.08	101.9	–480.1	1212	–1672	1184	–336.9
SS2-X−	1.779	–17.66	166.7	–737.4	1714	–2171	1418	–374.9
SS2-Y+	1.677	–10.05	92.2	–444.6	1146	–1609	1158	–334.7
SS2-Z−	1.731	–15.49	146.2	–663.5	1599	–2108	1434	–393.6

## Data Availability

The data used in this research is owned by *Instituto Universitario de Microgravedad* “*Ignacio Da Riva*” (*IDR*/*UPM*), *Universidad Politécnica de Madrid*. Any request regarding the data should contact this institution.

## Appendix A

The illumination testing, where the *I–V* curves were measured (see Figure 10), was carried out to assess the photodiode follows the 1-Diode/2-Resistor model. When the parameters of the model have been characterized (Table 2), the effect of the temperature can be derived from the model (Equation (1)) and the manufacturer data.

Assuming all parameters from Equation (1) are constant, except the short circuit current, *I_sc_* = *I*(*T*, *R_L_* = 0), and the photovoltaic current, *I_pv_*(*T*), the following equation can be obtained by deriving the aforementioned Equation (1):

(A1) $$dI_{sc}=\frac{\partial I_{pv}}{\partial T}dT-I_{0}\exp\left(\frac{qI_{sc}R_{s}}{a\kappa T}\right)\left(-\frac{qI_{sc}R_{s}}{a\kappa T^{2}}dT+\frac{qR_{s}}{a\kappa T}dI_{sc}\right)+\frac{R_{s}}{R_{sh}}dI_{sc},$$

which leads to:

(A2) $$\left(1+\frac{qI_{0}R_{s}}{a\kappa T}\exp\left(\frac{qI_{sc}R_{s}}{a\kappa T}\right)-\frac{R_{s}}{R_{sh}}\right)dI_{sc}=\left(\frac{\partial I_{pv}}{\partial T}+\frac{qI_{sc}I_{0}R_{s}}{a\kappa T^{2}}\exp\left(\frac{qI_{sc}R_{s}}{a\kappa T}\right)\right)dT.$$

From the parameters of the fitted model (see Table 2), the maximum irradiance in the orbit and the temperature range, it can be verified that:

(A3) $$\frac{qI_{0}R_{s}}{a\kappa T}\exp\left(\frac{qI_{sc}R_{s}}{a\kappa T}\right)\ll 1\;\;\;\;\;\;\frac{R_{s}}{R_{sh}}\ll 1.$$

Additionally, taking into account the change in the short circuit current given by the manufacturer [22], the following expression can be derived:

(A4) $$\frac{dI_{sc}}{dT}=\eta_{T}I_{sc}\approx \left(\frac{\partial I_{pv}}{\partial T}+\frac{qI_{sc}I_{0}R_{s}}{a\kappa T^{2}}\exp\left(\frac{qI_{sc}R_{s}}{a\kappa T}\right)\right).$$

When comparing the value given by the manufacturer with the right term from the above equation, it is possible to reach the following conclusion:

(A5) $$\eta_{T}\gg \frac{qI_{0}R_{s}}{a\kappa T^{2}}\exp\left(\frac{qI_{sc}R_{s}}{a\kappa T}\right),$$

which points out that the change in the photovoltaic current with the temperature is approximately the same as the change in the short circuit current:

(A6) $$\frac{\partial I_{pv}}{\partial T}\approx \frac{dI_{sc}}{dT}=\eta_{T}I_{sc}.$$

Bearing in mind that *I_pv_* ≈ *I_sc_*, it is possible to derive the following equation:

(A7) $$I_{pv}\left(T\right)=I_{pv,0}\exp\left(\eta_{T}\left(T-T_{0}\right)\right).$$

Assuming that the sensitivity of the photodiode scales equally in all the wavelengths, the change in the photovoltaic current, *I_pv_*, with the temperature can be rewritten in terms of the parameter *P* as:

(A8) $$P\left(T\right)=P_{0}\exp\left(\eta_{T}\left(T-T_{0}\right)\right),$$

where the subscript 0 indicates the value of the parameter at the reference temperature *T*_0_.

When expression (A8) is evaluated for a reference temperature of 20 °C and the extreme cold temperature from the in-flight readout of the nearest temperature sensor (−15 °C), the variation of parameter *P* is around −6%. This correction can be easily applied during the data post-processing. However, the magnitude is low enough, making the error assumable in the present work.
